# An observational study of anaerobic bacteria in cystic fibrosis lung using culture dependant and independent approaches

**DOI:** 10.1038/s41598-021-85592-w

**Published:** 2021-03-25

**Authors:** Claudie Lamoureux, Charles-Antoine Guilloux, Clémence Beauruelle, Stéphanie Gouriou, Sophie Ramel, Anne Dirou, Jean Le Bihan, Krista Revert, Thomas Ropars, Rosyne Lagrafeuille, Sophie Vallet, Rozenn Le Berre, Emmanuel Nowak, Geneviève Héry-Arnaud

**Affiliations:** 1 INSERM, EFS, Univ Brest, UMR 1078, GGB, 29200 Brest, France; 2 Department of Bacteriology, Virology, Hospital Hygiene, and Parasitology-Mycology, Brest University Hospital, Boulevard Tanguy Prigent, 29200 Brest, France; 3 Cystic Fibrosis Center of Roscoff, Fondation Ildys, Roscoff, France; 4 Department of Pulmonary and Internal Medicine, Brest University Hospital, Brest, France; 5 INSERM CIC 1412, Brest University Hospital, Brest, France

**Keywords:** Cystic fibrosis, Clinical microbiology

## Abstract

Strict anaerobes are undeniably important residents of the cystic fibrosis (CF) lung but are still unknowns. The main objectives of this study were to describe anaerobic bacteria diversity in CF airway microbiota and to evaluate the association with lung function. An observational study was conducted during eight months. A hundred and one patients were enrolled in the study, and 150 sputum samples were collected using a sterile sample kit designed to preserve anaerobic conditions. An extended-culture approach on 112 sputa and a molecular approach (quantitative PCR targeting three of the main anaerobic genera in CF lung: *Prevotella, Veillonella,* and *Fusobacterium*) on 141 sputa were developed. On culture, 91.1% of sputa were positive for at least one anaerobic bacterial species, with an average of six anaerobic species detected per sputum. Thirty-one anaerobic genera and 69 species were found, which is the largest anaerobe diversity ever reported in CF lungs. Better lung function (defined as Forced Expiratory Volume in one second > 70%) was significantly associated with higher quantification of *Veillonella*. These results raise the question of the potential impact of anaerobes on lung function.

## Introduction

Cystic fibrosis (CF) is a genetic disease due to CF transmembrane conductance regulator (*cftr)* gene mutations (mostly p.F508del mutation). From another point of view, CF can also be considered as an infectious disease, as lung colonisation and infection aggravate the clinical condition and are the main causes of morbidity-mortality in people with CF (PWCF). Key bacterial pathogens (*Staphylococcus aureus, Haemophilus influenzae*, *Pseudomonas aeruginosa* and *Burkholderia cepacia* complex) are known to chronically colonise CF lungs and cause pulmonary exacerbation^1^. However, CF pulmonary microbiota harbours a wider range of bacterial communities that include certain species thought to play an important role in respiratory disease^2^. An accurate description of the lung bacterial species, many of which have yet to be identified^3,4^, could lead to a better understanding of the lung disease^2^. Strict anaerobes play an important role among these as yet unknown species, and are undeniably important residents of the CF lung^5,6^, that have been detected in respiratory samples at all ages^4^. Anaerobes’ growth is stimulated by anoxic conditions, provided partially by mucus hyperviscosity and oxygen consumption by neutrophil afflux or bacterial proliferation^5,7^. The most frequently detected genera in CF lungs are *Prevotella, Veillonella, Fusobacterium, Atopobium, Peptostreptococcus* and *Porphyromonas*^4,5,8^. However, in spite of their wide diversity and abundance, the role of airway anaerobic bacteria is still controversial. On the one hand, anaerobes exhibit their own virulence factors such as proteases^9,10^, can enhance others pathogens virulence^11–14^ or can promote antibiotic resistance^15,16^. On the other hand, they can be associated with less lung inflammation^6,17^ and better lung function^4,6,18–20^. Hence, it remains under debate whether the presence of anaerobes is beneficial and whether targeted antibiotic therapy against anaerobic bacteria is needed or should rather be avoided in case of CF pulmonary exacerbation^4,21^. In the light of these contradictory observations, we conducted an observational study to go deeper in deciphering anaerobe diversity at species level within the CF lung microbiota. We implemented a dual approach, combining extended-culture and targeted molecular techniques for optimally exhaustive and accurate identification and quantification of anaerobic communities.

## Results

### Subjects and samples

**Figure 1:**
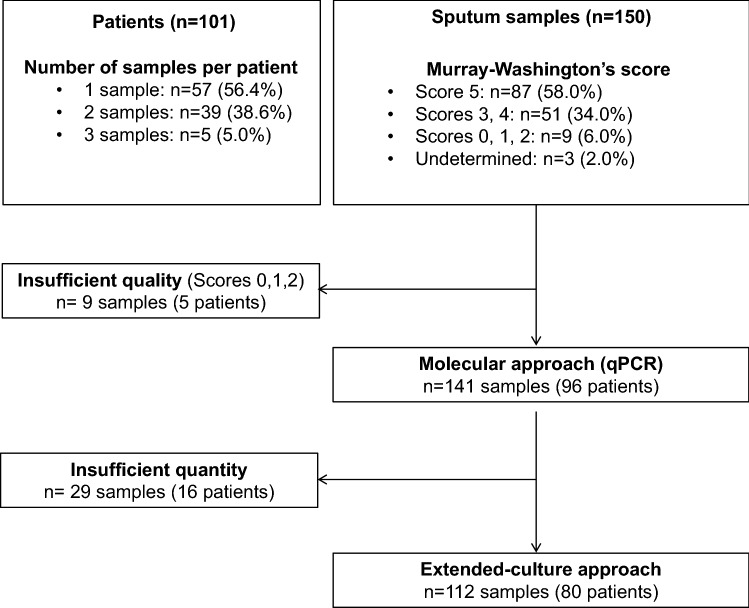
Flow chart of the patients and samples included in the study.

From March to October 2018, 150 sputa from 101 PWCF were collected in the study: one sample for 57 patients, and two or three samples for 54 patients. Based on Murray-Washington criteria^22,23^ (Table S1), salivary contamination was low in 58.0% of sputa (87/150), intermediate in 34.0% (51/150), and high in 6.0% (9/150); 2.0% of sputa (3/150) were undetermined because of poor quantity (Fig. 1). The nine samples with high salivary contamination were excluded from further analysis (five patients) and 29 sputa underwent molecular analyses but not extended-culture due to insufficient volume (16 patients) (Fig. 1). After exclusion, a total of 96 PWCF were enrolled in the study; subject characteristics are shown in Table 1 (and in Table S2 for the 80 PWCF whose samples were analysed only by extended-culture approach). According to the Leeds definition for *P. aeruginosa* colonisation^24^, the majority of the cohort were categorised as chronic or intermittent (72.9%; 70/96). Concerning lung function, 80.3% of patients (77/96) presented Forced Expiratory Volume in one second (FEV_1_) inferior or equal to 70%.

**Table 1:** Study cohort (96 people with cystic fibrosis): demographic and clinical data.

Patient characteristics	n (%)
**Age group (years)**	
< 13	7 (7.3)
13–< 18	2 (2.1)
18–< 25	30 (31.3)
25–< 30	14 (14.6)
≥ 30	43 (44.7)
**Gender**	
Female	47 (49.0)
Male	49 (51.0)
***cftr*** **mutation**	
p.F508del homozygote	53 (55.2)
p.F508del heterozygote	33 (34.4)
Other mutation	10 (10.4)
**Pancreas status**	
Pancreatic sufficiency	8 (8.3)
Pancreatic insufficiency	88 (91.7)
**Body-mass index**^**▲**^** (kg/m**^**2**^**)**	
Underweight (< 18.5)	22 (22.9)
Reference value (18.5–24.9)	63 (65.6)
Overweight/Obesity (> 24.9)	11 (11.5)
**Lung function (Forced Expiratory Volume in one second, %)**	
< 40	23 (24.0)
40–70	54 (56.3)
> 70	19 (19.7)
**Chronic antibiotic therapy (inhaled and/or oral)**	
Azithromycin	53 (55.2)
Tobramycin	19 (19.8)
Colistin	49 (51.0)
Aztreonam	8 (8.3)
**Oral antibiotic therapy (one month before)**	
Yes	39 (40.6)
No	57 (59.4)
**Corticosteroids (inhaled and/or oral)**	
Yes	73 (76.0)
No	23 (24.0)
**Diabetes**	
Yes	38 (39.6)
No	58 (60.4)
**CFTR modulators (ivacaftor, lumacaftor)**	
Yes	29 (30.2)
No	62 (64.6)
Change during study	5 (5.2)
**Leeds status (*Pseudomonas aeruginosa*** **colonisation)**	
Never	5 (5.2)
Free	21 (21.9)
Intermittent	11 (11.5)
Chronic	59 (61.4)

### Extended-culture approach for anaerobic bacteria isolation and identification at species level

**Figure 2:**
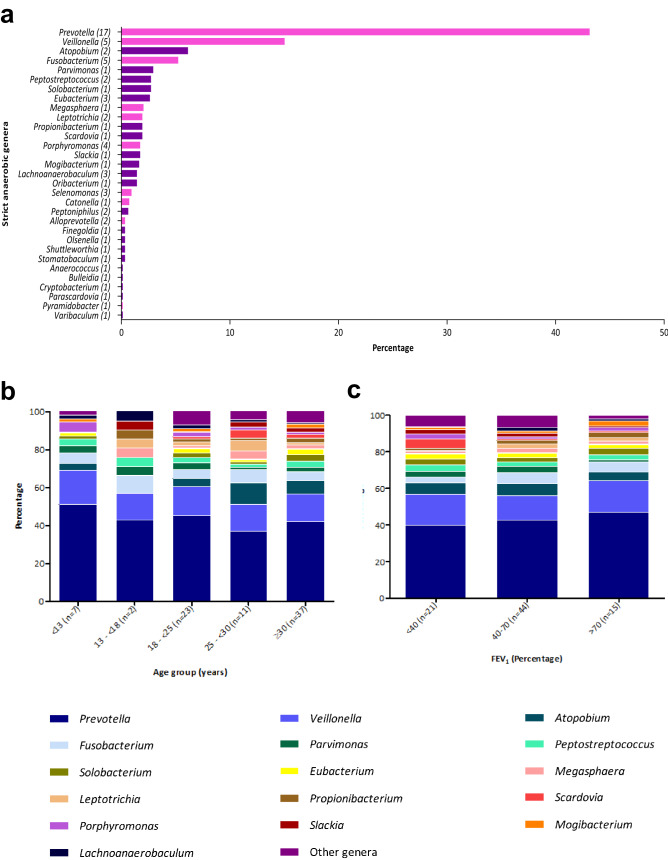
Extended-culture approach results. (**a**) Description of strict anaerobic genera identified in 112 sputum samples: Gram-positive genera are shown in purple, Gram-negative genera in pink, and number of species in brackets. (**b**) Prevalence of strict anaerobic genera identified according to patient age group (80 patients). (**c**) Prevalence of strict anaerobic genera identified according to patient lung function evaluated by Forced Expiratory Volume in one second (FEV_1_) (80 patients).

On the extended-culture approach, 692 strict anaerobic strains were isolated from the 112 sputa analysed: 84.8% were identified by MALDI-TOF MS (587/692), and 15.2% by 16S rRNA gene sequencing (105/692). A total of 91.1% of sputum samples (102/112) were positive for at least one anaerobic species. After the exclusion of duplicate species per patient (Table S3), an average of six anaerobic bacteria were isolated per patient (range 0–16). Thirty-one strict anaerobic genera were cultured from the 112 sputum samples, comprising 69 species (Fig. 2a; Table S4). The 10 most frequent genera were: *Prevotella* (43.1%; 298/692), *Veillonella* (15.0%;104/692), *Atopobium* (6.1%; 42/692), *Fusobacterium* (5.2%; 36/692), *Parvimonas* (2.9%; 20/692), *Peptostreptococcus* (2.7%; 19/692), *Solobacterium* (2.7%; 19/692), *Eubacterium* (2.6%; 18/692), *Megasphaera* (2.0%; 14/692), and *Leptotrichia* (1.9%; 13/692) (Fig. 2a). Anaerobic genera composition within the different age- and lung function- groups is detailed in Fig. 2b/2c. Two anaerobic genera, poorly represented but both isolated from high-quality sputum (Murray-Washington score at 5), were first detected in the CF lung: *Pyramidobacter* (0.1%; 1/692; species *Pyramidobacter piscolens*), and *Varibaculum* (0.1%; 1/692; species *Varibaculum anthropi*). At a finer taxonomic level, a wide diversity was found: 17 *Prevotella* species (*P. baroniae, P. buccae, P. denticola, P. histicola, P. intermedia, P. loescheii, P. maculosa, P. marshii, P. melaninogenica, P. nanceiensis, P. nigrescens, P. oralis, P. oulorum, P. pallens, P. salivae, P. timonensis,* and *P. veroralis)* five *Veillonella* species (*V. atypica, V. denticariosi, V. dispar, V. parvula,* and *Veillonella* sp.), and five *Fusobacterium* species (*F. canifelinum, F. naviforme, F. nucleatum, F. periodonticum,* and *Fusobacterium* sp.) (Table S4).

### Molecular approach for detection and quantification of *Prevotella*, *Veillonella* and *Fusobacterium* genera

qPCR targeting these three anaerobic genera in the lung confirmed that *Prevotella* was the most abundant genus in sputa (median of quantification: 1.34 × 10^6^ CFU/mL), compared to *Veillonella* (median of quantification: 2.71 × 10^4^ CFU/mL) and *Fusobacterium* (median of quantification: 2.55 × 10^2^ CFU/mL). The molecular approach was significantly more positive than culture for the detection of the less abundant genera, *Veillonella* and *Fusobacterium* (*p* < 0.01 respectively, McNemar test) (Table S5).

### Relationship between lung function and *Prevotella*, *Veillonella* and *Fusobacterium* quantification

Lung function was evaluated by FEV_1_, with a 70% clinical threshold^25^. Statistical analysis (univariate analysis) of results for the 96 patients (141 samples) showed that a one-log increase of *Prevotella*, *Veillonella* and *Fusobacterium* quantification (CFU/mL) was significantly associated with a FEV_1_ superior to 70% (OR = 1.41, *p* = 0.045; OR = 1.92, *p* < 0.001; OR = 1.45, *p* = 0.032 respectively). Multivariate analysis confirmed that a one-log increase of the quantity of the genus *Veillonella* was associated with a better lung function (FEV_1_ > 70%) (*p* = 0.02, logistic regression) (Table 2). Except for patient age, no other significant associations were found between lung function and the clinical parameters (gender, body-mass index, oral antibiotic therapy, CFTR modulator treatment, or *P. aeruginosa* positive sputum culture) (Table 2). Moreover, all 13 samples in which *Veillonella* was not detected by qPCR came from patients with FEV_1_ < 70% (10 patients, median age: 36 years). Focusing on the three most identified *Veillonella* species by culture approach (*V. atypica*, n = 34; *V. dispar*, n = 21; *V. parvula*, n = 47), *V. dispar* was the most associated with a better lung function (FEV_1_ > 70%) but this association was not significant (OR = 3.50, *p* = 0.08).

**Table 2:** Multivariate analysis: logistic regression modelling the probability of Forced Expiratory Volume in one second (FEV_1_) > 70%.

	OR^▲^ (95% confidence interval)	*p* value
Quantification of *Veillonella*^a^	2.14 (1.30–3.53)	< 0.01
Age^b^	0.86 (0.80–0.92)	< 0.01
Gender^c^	1.48 (0.42–5.19)	0.54
Body-mass index^d^	0.34 (0.11–1.03)	0.06
CFTR modulators^e^	1.07 (0.29–3.94)	0.92
Oral antibiotic treatment^e^	0.92 (0.23–3.62)	0.90
*Pseudomonas aeruginosa* positive culture^e^	0.56 (0.17–1.79)	0.33

^a^Odds-ratio relative to a one-log increase.

^b^Odds-ratio relative to a one-year increase.

^c^Woman *versus* man.

^d^ < 18.5 kg/m^2^ *versus* > 18.5 kg/m^2^.

^e^Yes *versus* no.

## Discussion

CF lungs harbour diverse communities of bacteria, the abundance and variety of which fluctuate depending on multiple factors: clinical status, treatment and environment^3,26–28^. Within this rich and diversified bacterial microbiota, anaerobes are able to grow in anoxic areas in CF lungs and have been detected in higher quantities in PWCF than in healthy individuals^5^. These oxygen-sensitive bacteria come from diverse reservoirs (mainly oral and digestive)^29^ and can be detected in equal or higher proportions than some CF pathogens in lung^4^. Moreover, numerous anaerobic genera have been identified as part of the CF core lung microbiota: *Prevotella, Veillonella, Porphyromonas, Fusobacterium, Catonella* and *Peptostreptococcus*^20,28,30^. The recent literature emphasised the importance of anaerobic bacteria in lung, but results and hypotheses concerning their role in the pathophysiology and progression of CF disease are contradictory^4,6,9–21,31,32^. Anaerobes’ impact is mainly evaluated according to genus rather than species, which may explain these conflicting results, as some species within a given genus may generate contrasting effects^33^. Thus, anaerobic microbiota description should be exhaustive, as accurate as possible and frequently implemented.

To this end, culture approaches seem to be appropriate to provide descriptive information of anaerobe diversity at species level^34^. However, due to the fastidiousness of implementing a strictly anaerobic atmosphere, lower sensitivity than molecular approaches, the effect of previous antibiotic therapy, and growth limited to “culturable” bacteria, most descriptive studies of the lung “anaerobiome” are currently provided by 16S-targeted metagenomics. However, the targeting of 16S variable regions with most of the sequencing platforms used did not allow the optimal taxonomic resolution to be achieved^35^. We decided to conduct an observational study, based on a dual approach combining extended-culture and targeted molecular techniques to go deeper in deciphering anaerobe diversity within the CF lung microbiota. To evaluate sputum quality, we used the Murray-Washington cytological score, which showed that 94.0% of the sputum samples (141/150) were properly sampled and informative. Our findings supplement previous culture data on anaerobe diversity^4–6,8^. Thanks to the innovative collecting device and the culture-extensive protocol (21 days of incubation) coupled with both MALDI-TOF MS and 16S rRNA gene sequencing, 31 strict anaerobic genera and 69 species were described in 112 CF sputa. Considering the seven studies that described strict anaerobes in the CF lung microbiota based on an extensive-culture approach (Table 3), our study provided the greatest diversity ever described so far. Mirkovic et al.^13^ found 52.3% positive respiratory samples with 8 anaerobic genera detected from 109 samples, Muhlebach et al*.*^4^ found 67.0% positive sputa with 18 anaerobic genera detected from 200 samples; O’Neill et al*.*^6^ found 12 genera from 41 sputum, Paganin et al*.*^36^ found five genera from 78 sputum, Sherrard et al*.*^37^ found 23 anaerobic genera from 199 sputa; Sibley et al*.*^38^ found 15 genera from 246 sputum, and Tunney et al*.*^5^ found 64.0% positive sputa with seven genera from 66 samples. Focusing on the most recent study which reported 18 anaerobic genera on culture in CF lung^4^, all of them were also detected in the present study, supplemented by description of 13 additional genera. To our knowledge, two anaerobic genera (*Pyramidobacter* and *Varibaculum*) were described here in CF lung for the first time in sputa with low salivary contamination. These culture results were contributive as they demonstrate the viability of anaerobes in CF lung, which might not be clear using molecular techniques, and they shed light on anaerobic communities in CF lung according to species. On the molecular side of the study, qPCR improved detection compared with culture for two anaerobic genera (*Veillonella* and *Fusobacterium*) which were represented at lower rates in sputum samples. These results showed that the combination of both approaches is essential for optimally exhaustive anaerobic microbiota description.

**Table 3:** Comparison of studies based on an extended-culture approach for the CF “anaerobiome” description.

References	Number of patients/samples (type of samples)	Clinical state	Taxonomic affiliation detailed to the genus/species level	Number of strict anaerobic genera/species/ isolates^b^	Delay between the sampling and anaerobiosis	Incubation time (days)	Identification methods applied on colonies
This study	80/112 (sputum)	All patients	Yes/yes	31/69/692	No delay	21	MALDI-TOF MS^c^, targeted qPCR and 16S rRNA gene sequencing
Sherrard et al.^37^	80/199 (sputum)	All patients	Yes/no	23/NR/NM	No delay	2–5	16S rRNA gene sequencing
Muhlebach et al.^4^	255/255 (sputum, BAL)	All patients	Yes/yes^a^	18/49/NM	No delay	2–7	16S rRNA gene sequencing
Mirković et al.^13^	109/109 (sputum, BAL)	Only stable patients	Yes/yes^a^	8/22/NM	No delay	5–7	16S rRNA gene sequencing
O’Neill et al.^6^	41/41 (sputum)	Only stable patients	Yes/no	12/NM/NM	No delay	5–7	16S rRNA gene sequencing
Paganin et al.^36^	78/78 (sputum)	All patients	Yes/yes	5/18/NM	15 min	5–7	MALDI-TOF MS and 16S rRNA/*rec*A-gene sequencing
Sibley et al.^38^	117/246 (sputum)	All patients	Yes/yes	15/28/157	2 min	7	16S rRNA gene sequencing
Tunney et al.^5^	60/76 (sputum, BAL)	Only stable patients	Yes/yes^a^	7/12/NM	15 min	5–7	16S rRNA gene sequencing and RapID Ana II identification system

*NR* not realized, *NM* not mentioned, *BAL* bronchoalveolar lavage fluid, *MALDI-TOF MS* matrix-assisted laser desorption/ionization time-of-flight mass spectrometry.

^a^Not available for all isolates.

^b^Only strict anaerobes, according to the Bergey’s Manual of Systematic Bacteriology definition^46^, were considered (if data available).

^b^Identification according to Li et al.^47^

As well as being descriptive, this study demonstrated a significant positive association between *Veillonella* quantification and lung function (FEV_1_ > 70%). This was already seen in previous studies, for the genus *Veillonella*^6^ and also for the genus *Prevotella*^18^. Moreover, although the observation was based on a small number of samples (n = 13), the genus *Veillonella* was absent only in sputa from PWCF with poor respiratory function (FEV_1_ < 70%). Consequently, the genus *Veillonella* and associated species detected on culture (*V. parvula, V. atypica, V. dispar)* could in future be used as biomarkers to predict lung function, as previously shown for the anaerobic genus *Porphyromonas* which could be used to predict earlier *P. aeruginosa* infection^39^. In our study, there is a tendency of association of the species *V. dispar* with a better lung function (non-significant association). The lung role of this species in PWCF is as yet unknown but a recent study in healthy people has shown that lung bacteria are the same as oral or nasal bacteria, but that some, like *V. dispar*, are enriched in the healthy lung^40^. This finding suggests that *V. dispar* may play an important role in lung health^40^, which needs further investigation. These results also pave the way for discussion of the protective role of anaerobic bacteria. However, little is known about the pathophysiology and mechanisms involved in this potential positive impact, although significant positive correlations were demonstrated between anaerobes and better lung function or/and less airway inflammation, especially for the genera *Prevotella, Veillonella* and *Porphyromonas*^1,4,6,18,20^.

The present study and the methods used here are subject to some limitations. Firstly, the cohort was composed of relatively old PWCF (median age, 27 years; only 12 patients under 18 years of age), which might constitute a recruitment bias for the description of anaerobic bacteria in CF lung, as diversity may be impacted by patient age^17,41^. Secondly, as the design was single-centre, geographical background was not taken into account, although it may have an impact on the richness and diversity of the anaerobic microbiota. Thirdly, molecular studies were performed only on three anaerobic genera (*Prevotella, Veillonella* and *Fusobacterium*). The anaerobe panel needs to be extended, and should target species rather than genera to accurately evaluate the impact of anaerobes on lung function. Fourthly, the Murray-Washington criteria were used to determine sputum quality and to exclude samples suspected of high salivary contamination. However this score does not make it possible to assert the only pulmonary location of the anaerobic bacteria detected. Fifthly, FEV_1_ may not be the most relevant lung parameter to evaluate lung function. Lung clearance index (LCI) was reported to show greater sensitivity than FEV_1_ and better correlation with clinical outcome. However, LCI is now indicated mainly for trials in young PWCF or with early or mild lung disease^42^, which were not the main conditions in our cohort. All in all, new longitudinal studies are required to better understand anaerobic microbiota dynamics and impact on respiratory function, depending on the patient’s clinical status, age (focusing on paediatric patients) and antimicrobial treatments, with inclusion in several CF centres.

In conclusion, this cohort study revealed the largest anaerobe diversity ever reported in CF lungs, thanks to preservation of anaerobic atmosphere in sputa and the powerful performance of the extended-culture approach. Moreover, the study highlighted that a greater quantity of the genus *Veillonella* was significantly positively associated with better lung function. Consequently, anaerobes should not be underestimated within the lung microbiota. Subsequent longitudinal studies are needed to determine and understand anaerobe impact and pathophysiology according to species and to evaluate anaerobe antibiotic sensitivity under antibiotic pressure.

## Methods

### People with cystic fibrosis (PWCF) and sample processing

This observational study was conducted between March and October 2018. Samples were collected in the Western Brittany CF centre (Roscoff, France) during follow-up consultations. As samples consisted of sputa, only PWCF able to expectorate were enrolled. Pulmonary transplant patients were excluded. The following sociodemographic and clinical parameters were recorded: age, gender, *cftr* gene mutation, pancreas status, body-mass index, lung function, diabetes, and treatments (oral antibiotic therapy one month before sampling, chronic antibiotic therapy, corticosteroids, CFTR modulators). A collecting device was specifically designed and patented for the study to ensure preservation of an anaerobic atmosphere for the sputa (EP 20305133.9) (Fig. S1). Between collection and transfer to the anaerobic chamber, samples were conserved at + 4 °C.

### Extended-culture approach for bacterial isolation and identification

Sputa quality and salivary contamination were evaluated on Murray-Washington score^22,23^ (Table S1). Each sputum sample was mixed with an equal volume of dithiothreitol (Digest-EUR, Eurobio, Courtaboeuf, France), and incubated at 37 °C for 30 min to one hour in accordance with the manufacturer’s instructions. One-thousand-fold dilution was prepared in physiological saline supplemented with 0.05% (wt/vol) L-cystein (Sigma-Aldrich, Dorset, UK). One hundred microliters of liquefied diluted sputum was plated onto three different media: anaerobe basal agar with sheep blood (ABA-SB) (ThermoFisher, Waltham, USA), kanamycin-vancomycin ABA-SB, and colistin-nalidixic acid ABA-SB. Plates were incubated in an anaerobic chamber (90% N_2_, 5% H_2_, 5% CO_2_) (Bactron, Sheldon Manufacturing, Cornelius, USA) for 21 days at 37 °C and readings were made every 2–5 days. Isolates of each distinct colony type were subcultured onto ABA-SB, and identified by matrix-assisted laser desorption/ionisation time-of-flight mass spectrometry (MALDI-TOF MS Biotyper MBT) (Bruker, Billerica, USA) (Supplementary Information). All strict anaerobes isolates were stored at − 80 °C (Microbank, Pro-Lab Diagnostics, Ontario, Canada). In case of mass spectrometry failure (database or technical problems), a 16S rRNA gene (*rrs*) sequencing method was used for isolate identification. The following primers were used for amplification of the *rrs* gene (996 bp): sense 5′-CCAGCAGCCGCCGTAATACG-3′; antisense 5′-TACGGYTACCTTGTTACGACT-3′. The PCR reaction was conducted using the Eppendorf Mastercycler Gradient instrument (Eppendorf, Hamburg, Germany). PCR sequencing used the Big Dye Terminator Cycle Sequencing Kit Ready Reaction version 3.1 (Applied Biosystems, Courtaboeuf, France), according to the manufacturer’s recommendations, on an Applied Biosytems 3130xl instrument (ThermoFisher, Waltham, USA). Sequences were then aligned and corrected using the software BioEdit version 7.0.5.3 (http://www.mbio.ncsu.edu/bioedit/bioedit.html). Comparison with the NCBI BLAST database (Basic Local Alignment Search Tool, http://blast.ncbi.nlm.nih.gov/Blast.cgi) enabled bacterial identification, with ≥ 98% similarity determining homology of identity. In parallel to the anaerobic extended-culture approach, all samples were cultured in aerobic conditions to detect usual pathogens such as *P. aeruginosa*.

### Detection and quantification of *Prevotella*, *Veillonella* and *Fusobacterium* genera by quantitative PCR (qPCR)

qPCR was performed for detection and quantification of three anaerobic genera (*Prevotella*, *Veillonella, Fusobacterium*) in each sputum sample. 200 µL aliquots of liquefied sputum were stored at − 80 °C. Samples were treated by five minutes-sonication using a bath sonicator (Elmasonic S10, Elma Schmidbauer GmbH, Singen, Germany). After 10 min-centrifugation (5000 g) and prior lysis by proteinase K, total DNA was extracted using the QIAamp DNA Minikit (Qiagen, Courtaboeuf, France) according to the manufacturer’s instructions, with an elution volume of 100 µL. The qPCR reaction was conducted using an ABI Prism 7500 Fast Real-Time PCR System instrument (ThermoFisher, Waltham, USA). Primers (and probe) used for qPCR are detailed in Table S6^43–45^. Specificity and efficiency were evaluated for each primer and probe pairs (Supplementary Information).

### Statistical analysis

McNemar’s test was used to compare the percentage of positive samples according to culture and molecular approaches. A logistic regression was used for binary outcome (FEV_1_ > 70%) to estimate odds-ratios relative to a one-log increase of the quantification of the anaerobic genera and to the absence/presence of *Veillonella* species (*V. atypica, V. dispar, V. parvula*) in univariate analysis. Only the most associated genus (*Veillonella*) and species (*V. dispar*) were considered in the multivariate model, adjusting for potential confounders (age, gender, body-mass index, CFTR modulators, oral antibiotic treatment and *P. aeruginosa* positive culture). Intra-patient correlation due to repeated samples was handled thanks to an exchangeable correlation structure, using the SAS software GENMOD procedure (SAS Institute, Cary, USA) with binomial distribution and logit link function.

### Ethics

The study was approved by the French Ethical Research Committee in March 2018 (2018-A00624-51). All experiments were performed in accordance with relevant guidelines and regulations. Informed consent was obtained from all participants and/or their legal guardians.

### Conference presentation

The study was presented in part at the 39th “Réunion Interdisciplinaire de Chimiothérapie Anti-Infectieuse-RICAI”, 16–17 December 2019 in Paris, France.

## Supplementary Information

Supplementary Information
