# Supplementary material for: An observational study of anaerobic bacteria in cystic fibrosis lung using culture dependant and independent approaches
Source: Sci Rep. 2021 Mar 25;11:6845. doi: 10.1038/s41598-021-85592-w (PMC7994387; doi:10.1038/s41598-021-85592-w)
Supplement: Supplementary file 1 — Supplementary Information [file 41598_2021_85592_MOESM1_ESM.pdf]

An observational study of anaerobic bacteria in cystic fibrosis lung using culture dependant and independent approaches

Claudie Lamoureux[1,2], Charles-Antoine Guilloux[1], Clémence Beauruelle[1,2], Stéphanie Gouriou[1], Sophie Ramel[3], Anne Dirou[3], Jean Le Bihan[3], Krista Revert[3], Thomas Ropars[3], Rosyne Lagrafeuille[1], Sophie Vallet[1,2], Rozenn Le Berre[1,4], Emmanuel Nowak[5], Geneviève Héry-Arnaud[1,2*]

[1)] Univ Brest, INSERM, EFS, UMR 1078, GGB, F-29200 Brest, France

[2)] Department of Bacteriology, Virology, Hospital Hygiene, and Parasitology-Mycology, Brest University Hospital, Brest, France

[3)] Cystic Fibrosis Center of Roscoff, Fondation Ildys, Roscoff, France

[4)] Department of Pulmonary and Internal Medicine, Brest University Hospital, Brest, France

[5)] INSERM CIC 1412, Brest University Hospital, Brest, France

Corresponding author:

Prof. Geneviève Héry-Arnaud, Department of Bacteriology-Virology, Hospital Hygiene, Parasitology-Mycology, Brest University Hospital, Boulevard Tanguy Prigent, 29200 Brest, France

Mail: hery@univ-brest.fr

Tel: +33 2 98 14 51 02/ Fax: +33 (0)2 98 14 51 49

Supplementary Information

Sample kit

The kit consists of a sterile polypropylene tube with a filter cap for gas exchange and an anaerobic atmosphere generator to reduce the oxygen content (<1% in 30 minutes), placed in a sealed plastic transport pouch closed with a removable bar (Fig. S1).

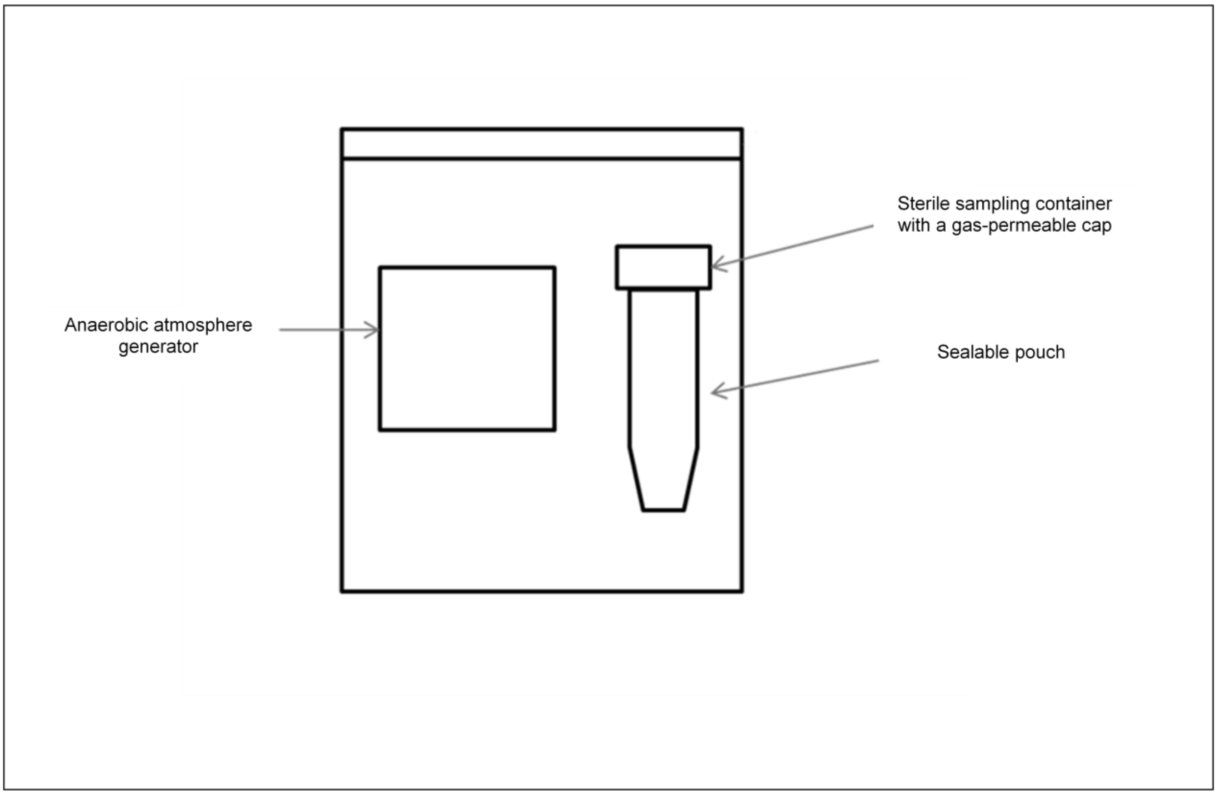

Figure S1. Sampling kit used in the study as described in the patent EP 20305133.9

Validation of qPCR assays targeting Prevotella, Veillonella *and* Fusobacterium

Molecular tests were performed to determine sensitivity, specificity and efficiency of our molecular approach (in addition to the initial assessment of primers [43–45]).

Sensitivity: several species have been selected to test the qPCR sensitivity (***Prevotella***: *P. buccae, P. buccalis, P. bivia, P. denticola, P. histicola, P. intermedia, P. maculosa, P. melaninogenica, P. nanceinsis, P. nigrescens, P. pallens, P. salivae, P. timonensis, P. veroralis;* ***Veillonella****: V. atypica, V. dispar, V. parvula;* ***Fusobacterium****: F. gonidiaformans, F. nucleatum, F. ulcerans).*

Specificity: a panel of bacteria and fungi was used to determined specificity of each primers Our choice was guided to include a wide variety of bacteria and fungi which are frequently detected in CF sputum (**Gram positive bacteria**: *Actinomyces odontolyticus, Gemella morbillorum, Granulicatella adiacens, Lactobacillus plantarum, Peptostreptococcus anaerobius, Rothia dentocariosa, Staphylococcus aureus, Streptococcus salivarius;* **Gram negative bacteria**: *Achromobacter xylosoxidans, Bacteroides fragilis, Burkholderia cepacia, Burkholderia multivorans, Campylobacter jejuni, Capnocytophaga sp, Fusobacterium nucleatum, Haemophilus parainfluenzae, Klebsiella pneumoniae, Neisseria flavescens, Porphyromonas gingivalis, Prevotella bivia, Pseudomonas aeruginosa, Pseudomonas putida, Stenotrophomonas maltophilia, Veillonella parvula;* **Mycobacteria**: *Mycobacterium abscessus;* **Fungi**: *Aspergillus sp, Candida albicans, Penicillium sp*).

Among the 28 microorganisms tested, five were detected by primers targeting *Prevotella*, three by primers targeting *Veillonella* and four by primers targeting *Fusobacterium*. All these species were detected positive with a Ct greater than 34. In order to compensate for this lack of specificity, a cut-off for Ct of 34 was determined for each qPCR; beyond this cut-off, the sample was considered negative.

Efficiency: the efficiency of each primer pair was determined, using a concentration range of pure and diluted DNA at 1/10th, 1/100th, 1/1000th and 1/10,000th. Each dilution was distributed in duplicate. Efficiencies of qPCR have been determined at 85.5% (R^2=1) for *Prevotella*, 92.6% (R^2=0.998) for *Veillonella*, and 88.2% (R^2 = 0.999) for *Fusobacterium*.

Interpretation of qPCR data

A concentration range from 10^2 to 10^8 CFU/mL, using a standard agar plate colony counting method, was achieved. Three species mainly represented for each genus were chosen (*Prevotella melaninogenica, Veillonella parvula,* and *Fusobacterium nucleatum*). Each concentration point of the range was extracted and a qPCR was performed to obtain a standard curve for each species.

Isolates identification by mass spectrometry (MALDI-TOF MS)

Culture isolates identification was performed using MALDI-TOF MS Biotyper MBT with the Bruker's library version 7 (Bruker, Billerica, USA) including 7311 reference spectra. This database references 2509 bacterial species, including nearly 300 strict anaerobic species. The identification criteria of

MALDI-TOF MS were as follows: a score of ≥2.0 was considered as accurate species-level identification; ≥ 1.7 but < 2.0 as a probable genus-level identification; an isolate with a score < 1.7 was considered as "unidentified" [47]. In order to improve isolates identification, 1 µL of 70% formic acid LC/MS (VWR, Radnor, USA) was added before the addition of 1 µL of portioned IVD-HHCA matrix (Bruker, Billerica, USA).

Table S1. Description of the Murray-Washington cytological score used for the sputum quality and salivary contamination appreciation (adapted to Bartlett's criteria for interpretation [23])

Score	Leucocytes number	Epithelial cells number	Salivary contamination
5	>25	<10	Low
4	>25	10-25	Intermediate
3	>25	>25	
2	10-25	>25	High
1	<10	>25	
0	<10	<10	

Table S2. Study cohort analysed only by extended-culture approach (80 people with cystic fibrosis): demographic and clinical data

Patient characteristics		n (%)
**Age group**	<13	7 (8.8)
(years)	13 - <18	2 (2.5)
	18 - <25	23 (28.7)
	25 - <30	11 (13.8)
	≥ 30	37 (46.2)
**Gender**	Female	39 (48.8)
	Male	41 (51.2)
***cftr* mutation**	p.F508del homozygote	43 (53.7)
	p.F508del heterozygote	27 (33.8)
	Other mutation	10 (12.5)
**Pancreas status**	Pancreatic sufficiency	7 (8.8)
	Pancreatic insufficiency	73 (91.2)
**Body-mass index**▲	Underweight (<18.5)	19 (23.8)
(kg/m^2)	Reference value (18.5-24.9)	53 (66.2)
	Overweight/Obesity (>24.9)	8 (10.0)
**Lung function**	<40	21 (26.3)
(Forced Expiratory Volume in one second, %)	40-70	44 (55.0
	>70	15 (18.7)
**Chronic antibiotic therapy**	Azithromycin	44 (55.0)
(inhaled and/or oral)	Tobramycin	17 (21.3)
	Colistin	40 (50.0)
	Aztreonam	8 (10.0)
**Oral antibiotic therapy**	Yes	34 (42.5)
(one month before)	No	46 (57.5)
**Corticosteroids**	Yes	60 (75.0)
(inhaled and/or oral)	No	20 (25.0)
**Diabetes**	Yes	32 (40.0)
	No	58 (60.0)
**CFTR modulators**	Yes	21 (26.3)
(ivacaftor, lumacaftor)	No	54 (67.5
	Change during study	5 (6.2)
**Leeds status**	Never	5 (6.2)
(*Pseudomonas aeruginosa* colonisation)	Free	18 (22.5)
	Intermittent	9 (11.3)
	Chronic	48 (60.0)

Table S3. Number of duplicate and non-duplicate species per patient (by extended-culture approach)

Patient identification (number of samples)	Total number of duplicate species	Total number of non-duplicate species	Percentage of duplicate species per patient
P2 (2)	4	17	19.0
P4 (2)	0	9	0
P5 (2)	4	8	33.3
P12 (2)	4	16	20.0
P14 (2)	2	10	16.7
P17 (3)	7	19	26.9
P18 (3)	8	16	33.3
P24 (2)	0	2	0
P37 (2)	2	12	14.3
P40 (2)	4	19	17.4
P44 (2)	3	7	30.0
P45 (2)	3	16	15.8
P46 (2)	1	10	9.1
P57 (2)	2	7	22.2
P58 (2)	4	12	25.0
P59 (2)	3	16	15.8
P60 (3)	7	18	28.0
P61 (2)	1	7	12.5
P62 (2)	6	16	27.3
P63 (2)	0	11	0
P66 (2)	4	15	21.1
P67 (2)	3	12	20.0
P73 (2)	3	10	23.1
P76 (2)	7	21	25.0
P77 (2)	1	14	6.7
P84 (2)	2	12	14.3
P86 (2)	5	10	33.3
P87 (2)	0	2	0
P90 (2)	2	13	13.3

Table S4. List of the 69 species detected by extended culture in 112 CF sputum samples

Species	Percentage (n)
Alloprevotella rava ▲	0.1 (1)
Alloprevotella tannerae ▲	0.1 (1)
Anaerococcus murdochii o	0.1 (1)
Atopobium parvulum o	4.5 (31)
Atopobium rimae o	1.6 (11)
Bulleidia extructa o	0.1 (1)
Catonella morbi ▲	0.7 (5)
Cryptobacterium curtum ▲	0.1 (1)
Eubacterium brachy o	0.7 (5)
Eubacterium infirmum ▲	0.3 (2)
Eubacterium sulci ▲	1.6 (11)
Finegoldia magna o	0.3 (2)
Fusobacterium canifelinum o	0.1 (1)
Fusobacterium naviforme o	0.3 (2)
Fusobacterium nucleatum o ▲	3.7 (25)
Fusobacterium periodonticum o ▲	0.6 (5)
Fusobacterium sp. o ▲	0.4 (3)
Lachnoanaerobaculum orale o	0.9 (6)
Lachnoanaerobaculum umeaense o	0.3 (2)
Lachnoanaerobaculum sp. o ▲	0.3 (2)
Leptotrichia wadei o ▲	1.0 (7)
Leptotrichia sp. o▲	0.9 (6)
Megasphaera micronuciformis o	2.0 (14)
Mogibacterium diversum ▲	1.6 (11)
Olsenella uli o	0.3 (2)
Oribacterium sp. ▲	1.0 (7)
Parascardovia denticolens o	0.1 (1)
Parvimonas micra o	2.9 (20)
Peptoniphilus harei o	0.4 (3)
Peptoniphilus lacrimalis o	0.1 (1)
Peptostreptococcus anaerobius o	0.3 (2)
Peptostreptococcus stomatis ▲	2.5 (17)
Porphyromonas catoniae o ▲	0.3 (2)
Porphyromonas gingivalis o	0.1 (1)
Porphyromonas pasteri ▲	1.0 (7)
Porphyromonas uenonis o ▲	0.3 (2)
Prevotella baroniae o	0.1 (1)
Prevotella buccae o	1.4 (10)
Prevotella denticola o	3.5 (24)
Prevotella histicola o	5.9 (41)
Prevotella intermedia o	0.6 (4)
Prevotella loescheii o	0.3 (2)
Prevotella maculosa o	0.4 (3)
Prevotella marshii o	0.3 (2)

Prevotella melaninogenica °	9.5 (66)
Prevotella nanceiensis °	2.5 (17)
Prevotella nigrescens °	5.3 (37)
Prevotella oralis °	1.3 (9)
Prevotella oulorum °	1.0 (7)
Prevotella pallens °	3.6 (25)
Prevotella salivae °	5.8 (40)
Prevotella timonensis °	0.6 (4)
Prevotella veroralis °	0.9 (6)
Propionibacterium acidifaciens ° ▲	1.9 (13)
Pyramidobacter piscolens ▲	0.1 (1)
Scardovia wiggsiae ▲	1.9 (13)
Selenomonas infelix °	0.3 (2)
Selenomonas noxia ▲	0.1 (1)
Selenomonas sputigena °	0.4 (3)
Shuttleworthia satelles ▲	0.3 (2)
Slackia exigua °	1.7 (12)
Solobacterium moorei °	2.7 (19)
Stomatobaculum longum ▲	0.3 (2)
Varibaculum anthropi ▲	0.1 (1)
Veillonella atypica °	4.9 (34)
Veillonella denticariosi °	0.1 (1)
Veillonella dispar °	3.0 (21)
Veillonella parvula °	6.8 (47)
Veillonella sp. ▲	0.1 (1)

° identification by matrix-assisted laser desorption/ionization time-of-flight mass spectrometry (MALDI-TOF MS, Bruker)

▲identification by 16S rRNA gene sequencing

Table S5. Comparison of detection of three anaerobic genera (*Prevotella, Veillonella* and *Fusobacterium*) by extended-culture and targeted molecular (quantitative PCR) approaches in 112 sputum samples (McNemar test)

	**Culture approach**	**Molecular approach**		
	Percentage of positive samples (n)	Percentage of positive samples (n)	Median of quantification (CFU/mL)	***p*-value**
Prevotella	92.0 (103)	91.1 (102)	1.65×10^{6}	1
Veillonella	73.2 (82)	89.2 (100)	3.17×10^{4}	<0.01
Fusobacterium	32.1 (36)	67.9 (76)	1.12×10^{3}	<0.01

Table S6. Primers (and probe) used for detection and quantification of the genera *Prevotella*, *Veillonella* and *Fusobacterium* by quantitative PCR in sputum samples

Genus	Sequence*	Targeted gene	Reference
Prevotella	Forward : 5’-GATGCGTCTGATTAGCTT-3’<br>Reverse: 5’-CCAATATTCCTCACTGCTG-3’	16S	[44]
Veillonella	Forward : 5’-CCGTGATGGGATGGAAACTGC-3’<br>Reverse : 5’-CCTTCGCCACTGGTGTTCTTC-3’	16S	[43]
Fusobacterium	Forward : 5’-CGCAGAAGGTGAAAGTCCTGTAT-3’<br>Reverse : 5’-TGGTCCTCACTGATTCACACAGA-3’<br>Probe : 5’-FAM-ACTTTGCTCCCAAGTAACATGGAACACGAG-TAMRA-3’	16S	[45]

*FAM = 6-carboxyfluorescein; TAMRA = carboxytétraméthylrhodamine
